# Complete genome sequence of *Ancylobacter* sp. strain SB2, a hydrogen-oxidizing bacterium isolated from an alkaline mineral water spring

**DOI:** 10.1128/mra.00889-26

**Published:** 2026-10-27

**Authors:** Sijia He, Qi Wei, Takeshi Hayashi, Hajime Kobayashi

**Affiliations:** 1 Department of Systems Innovation, Graduate School of Engineering, the University of Tokyo 13143, Tokyo, Japan; 2 Department of Regional Studies and Humanities, Faculty of Education and Human Studies, Akita University 12712, Akita, Japan; 3 Frontier Research Center for Energy and Resources (FRCER), Graduate School of Engineering, the University of Tokyo 13143, Tokyo, Japan; California State University San Marcos, San Marcos, California, USA

**Keywords:** *Ancylobacter*, hydrogen-oxidizing bacteria, chemolithoautotrophy, alkaline environment

## Abstract

We report the complete genome sequence of *Ancylobacter* sp. strain SB2, a hydrogen-oxidizing bacterium isolated from an alkaline mineral water spring. The genome comprises 5,124,764 bp with 66.36% G + C content. Genome-based taxonomic analyses indicated strain SB2 is genomically distinct from the type strains of the *Ancylobacter* species examined.

## ANNOUNCEMENT

Hydrogen-oxidizing bacteria (HOB) have attracted interest as biocatalysts for CO_2_-based biomanufacturing (1). Most characterized HOB have been isolated from circumneutral environments, whereas those inhabiting alkaline environments remain largely underexplored (2). Members of the genus *Ancylobacter* are aerobic α-proteobacteria commonly found in aquatic and terrestrial environments (3), and several species, including *A. novellus*, are known to be HOB (4). Here, we report the complete genome sequence of *Ancylobacter* sp. strain SB2, the HOB isolated from an alkaline mineral water spring. A 500-mL water sample (pH 9.8) was collected from the Atago mineral spring (Akita, Japan; 39.15° N, 140.50° E) in June 2023, and a 0.1-mL aliquot was inoculated into a 50-mL batch of minimal medium (5) buffered with HCO₃^−^/CO₃²^−^ (pH 9.8). For enrichment, the culture was incubated under H₂/O₂/CO₂ (80:10:10) atmosphere at 25°C with agitation at 200 rpm. After three subcultures, the enrichment culture was streaked onto the same medium solidified with 1.5% (wt/vol) agar and incubated under the same atmosphere, yielding strain SB2.

Strain SB2 was propagated in 20 mL of tryptic soy broth at 25°C for 2 days and harvested by centrifugation. Genomic DNA was extracted using the Genomic-tip 20/G Kit (Qiagen) with the Genomic DNA Buffer Set (Qiagen), purified using DNA Clean Beads (MGI Tech) and DNeasy PowerClean Pro Cleanup Kit (Qiagen), and sheared into approximately 10–20-kb fragments with a g-TUBE (Covaris). Bioengineering Lab. Co., Ltd. (Kanagawa, Japan) prepared the sequencing library from 50 ng of the resulting DNA using the SMRTbell gDNA Sample Amplification Kit and SMRTbell Express Template Prep Kit 2.0 (PacBio), and performed sequencing on the PacBio Revio platform using the Revio Polymerase Kit (PacBio), according to its standard protocols. SMRT Link v.13.0.0.207600 was used to trim overhang adapter sequences and obtain HiFi reads. lima v.2.7.1 (https://github.com/pacificbiosciences/barcoding) trimmed the ultra-low PCR adapter sequences. pbmarkdup v.1.0.3 (https://github.com/PacificBiosciences/pbmarkdup) removed PCR duplicates, yielding 24,688 reads with a mean length of 9,961 bp. After excluding reads <1,000 bp and retaining the highest-scoring reads accounting for 90% of the total input bases using Filtlong v.0.2.1 (https://github.com/rrwick/Filtlong), the remaining 21,405 reads, with a mean length of 10,340 bp, were assembled using Flye v.2.9.2-b1786 (6). Genome circularity was confirmed using Bandage v.0.8.1 (https://github.com/rrwick/Bandage) (7), and genome completeness and contamination were assessed with CheckM2 v.1.0.1 (https://github.com/chklovski/CheckM2) (8), indicating 99.99% completeness and 0.46% contamination. The assembly comprised one circular chromosome (5,062,976 bp) and one circular plasmid pSB2 (61,788 bp), with 47.98× coverage and 66.36% G + C content.

Structural and functional annotation of the genome was performed using DFAST v.1.4.0 (https://dfast.ddbj.nig.ac.jp/) (9), identifying 4,816 protein-coding sequences, including genes encoding two [NiFe]-hydrogenases and enzymes involved in the Calvin–Benson–Bassham cycle, 2 rRNA operons, and 54 tRNAs. Genome-based taxonomic analysis using the Type (Strain) Genome Server (TYGS) (10) placed strain SB2 closest to *A. novellus* ATCC 8093 (GCA_000092925), with a digital DNA-DNA hybridization (dDDH) value of 25.2% and an average nucleotide identity (ANI), calculated by EzBioCloud calculator (https://www.ezbiocloud.net/tools/ani) (11), of 82.28%. Default parameters were used for all software, unless otherwise specified.

## Data Availability

The genome project is indexed at the DNA Data Bank of Japan (DDBJ) under the BioProject accession number PRJDB42576 and the BioSample accession number SAMD01933447. The complete genome sequence of *Ancylobacter* sp. strain SB2 is available in the DDBJ/EMBL/GenBank databases under the accession numbers AP050811 (chromosome) and AP050812 (plasmid pSB2). Raw reads can be found under the Sequence Read Archive with accession number DRR1066995.
